# Care homes and primary care in England working together: A multi-method qualitative study

**DOI:** 10.1177/13558196241306607

**Published:** 2024-12-13

**Authors:** Krystal Warmoth, Alex Aylward, Claire Goodman

**Affiliations:** 1 Senior Research Fellow, Centre for Research in Public Health and Community Care, 3769 University of Hertfordshire, Hatfield, UK; 2 Patient and Public Involvement Representative, Peninsula Public Involvement Group, 574931 National Institute of Health and Care Research Applied Research Collaboration, South West Peninsula, Exeter, UK; 3 Professor of Health Care Research, Centre for Research in Public Health and Community Care, 3769 University of Hertfordshire, Hatfield, UK

**Keywords:** long-term care, general practice, theory of change

## Abstract

**Objective:**

In England, most long-term care for older people with complex health care needs is provided by private care homes. They rely on primary care to provide medical care and access to specialist health care services. This study explored the working relationships between care homes and primary care in one region in England to inform a theory of change for achieving improved relationships.

**Methods:**

We carried out a multi-method qualitative study using appreciative inquiry. We thematically analysed data from 33 survey responses, 15 interviews, and eight workshops with care home and primary care staff, family carers, and other community specialists to populate the theory of change. A patient and public involvement representative supported data collection, analysis, and write-up.

**Results:**

Study participants described activities that encouraged role understanding, communication, and learning together benefitting staff, relationships, and quality of services. The lessons and experiences from the COVID-19 pandemic had shaped participants’ understanding of what is required to sustain cross-sector collaboration. Key inputs included time, staff, and funding to facilitate learning how to work together effectively, as well as the capacity to adapt to diverse care settings and address the complex, individual needs of care home residents. Participants noted the few opportunities they had to share their learning and discuss best practice.

**Conclusion:**

The theory of change identified different dimensions of good practice, providing insight into areas for action to inform service design and practice. Ongoing organisational changes should consider what is already working well and build on these achievements to enable positive care home and primary care working relationships and so foster high quality care and equitable access to services.

## Introduction

Globally, integration and collaboration between health and long-term care are encouraged by policymakers,^1^ including in England,^2^ where initiatives are being rolled out to improve health and care provision for people living in care homes.^3^ Long-term care for older people in England is largely provided by private care home providers. Most residents are over 85 years of age and live with complex health care needs, such as dementia and palliative care.^4^ Care home staff initiate and negotiate access to primary health care on behalf of their residents^5^; general practitioners (GPs) act as the leaders and coordinators of residents’ medical care.^3^

The COVID-19 pandemic exposed fragmentation between health and care home settings in England.^6^ Care homes often relied on individual health care professionals’ (namely GPs’) discretion about how and when they would visit, with existing strength and quality of the relationships between care homes and GPs often determining the care home staff’s capacity to respond to the pandemic.^7^ Post-pandemic, some GPs have reduced their face-to-face contact with care homes by shifting to remote working, using assistive technologies to support virtual monitoring and relying on care home staff to alert them to changes in residents’ health.^8^

The Enhanced Health Care in Care Homes (EHCH) framework, rolled out across England in 2020, was meant to strengthen collaboration and reduce known inequities in care home residents’ access to health care.^3^ This was meant to be achieved through the involvement of a consistent multidisciplinary team of health care professionals that includes specialist nurse practitioners or advanced clinical practitioners,^9^ a named GP, regular medicine reviews, ‘home rounds’, and personalised care plans. However, the evidence so far suggests that enhanced collaboration has been challenging, with one study documenting difficulties around the recruitment of specialist nurses, lack of experience in working with care homes, and role duplication.^10^

How GPs work with care homes is often determined by local care models, customs, and practices,^11^ for example, whether there are single or multiple GP practices working with the local care homes.^12^ Primary care teams often receive little recognition for work in care homes and there remains a lack of understanding of the nature of the resources that GPs need to work with care homes, or how to facilitate positive working relationships for health care and care home staff.^13^ There is some evidence suggesting that when GPs and other visiting health care professionals are actively supported with their work with older people in care homes, they consider staff as colleagues and experience lower callouts for emergency and urgent care.^14^

Much emphasis has been placed on making it easier for National Health Service (NHS) staff to work with care homes and create less demand for NHS services.^15^ However, whether policies and initiatives have improved the experiences of those working and living in care homes is not well understood. This study aimed to contribute to closing this evidence gap by exploring how GPs and care homes’ staff could be supported to improve appropriate access to health care. Our ultimate aim was to develop a Theory of Change (ToC) for the working relationship between care homes and primary care in England. The ToC can inform how high-quality primary care for people who live and work in and with care homes might be achieved and may be used to design health and social care services for older people.

## Methods

### Study design

We conducted a multi-method study using qualitative methods. Data collection focused on building a contextualised description of how study participants (see below) worked together and likely causal relationships that supported residents’ access to health care.^16^ Particular attention was given to participants’ activities, residents’ needs and priorities, and how different factors and conditions (e.g. different ways of working) were seen as enabling and sustaining care home residents’ access to services.

Data collection and analysis drew on the principles of appreciative inquiry. Appreciate inquiry is an organisational development method that seeks to identify strengths and positive aspects within a system to inspire collaborative problem-solving and drive sustainable change. It draws from action research, organisational learning, and organisational change. It offers a strength-based approach to organisational learning and development,^17^ aiming to foster collective identity and purpose,^18^ thereby helping to address some of the well-documented power differentials between health and social care settings.^19^ Appreciative inquiry follows a 4D cycle which is a four-step process that helps people, teams, and organisations improve by focusing on their strengths and successes. The phases are Discovery (learning from stories of best practice), Dream (what participants want more of), Design (participants’ priorities and ideas), and Destiny (how to put in place to make it happen).^17^ It provides a basis for a theory of what is likely to work and to improve staff and resident working relationships and older peoples’ health care-related outcomes.

### Data collection

We synthesised data from three data sources: (i) an online survey of GP practice staff about models of care and relationships with care homes,^20^ (ii) semi-structured interviews with care home and primary care staff, and (iii) participatory workshops with care home staff, family carers, GPs, and other community specialists. Data were collected between February 2022 to February 2023.

The online survey is described in detail elsewhere.^21^ In brief, we used Thiscovery, an online research platform. Questions focused on GPs’ work with care homes, with free text options for participants to share opinions on or experiences of what works well or improves appropriate access to services. Responses were mapped by topic and organised to reflect recurring issues and ways of working.^22^

Survey findings guided recruitment for and content of semi-structured interviews with care home and primary care staff. The survey identified five models of care: (1) care home has a named GP and pharmacist; (2) care home has a named GP and nurse practitioner; (3) care home is aligned to the GP surgery but has no named GP; (4) care home has a named GP and visiting paramedic; and (5) care homes have the same GP.^20^ We purposively selected five GP practices that had responded to the survey and that represented one of the five models of care. Selected practices were based in four different counties across the East of England region.

We then invited GP practices and the care homes that they supported (ranging from one to six) for an interview, obtaining consent and collecting demographic information using Thiscovery. Participants could schedule a telephone or video call interview with a female researcher with more than 10 years experience (KW) or self-record their responses to the interview questions online using Thiscovery. Participants were asked about their experiences of what worked well during the pandemic and how to improve care home and primary care working (Interview topic guide is shown in the Online Supplement). We conducted interviews with 10 primary care staff from four GP surgeries and five care home staff from four care homes participated in interviews. One participant self-recorded their responses to the interview questions online. Interviews lasted an average of 39 minutes.

Finally, we held eight online workshops and structured consultations with a convenience sample of care home managers, deputy managers, or clinical leads, nurses, GPs, primary care specialists, nurse practitioners, family carers, and visiting occupational therapists in November and December 2022; each workshop or consultation comprised 1-3 participants. Participants were recruited through emails promoting the study with supporting information through Clinical Research Networks, the National Institute for Health and Care Research’s Enabling Research in Care Homes network, and other care home and primary care representative groups (e.g. British Society of Gerontology Care Homes Research Special Interest Group). Interested participants were sent dates and times for the workshop and a link for the video call, with an information sheet and consent form to be returned before the workshop. Sixteen participants included care home managers, deputy managers, or clinical leads, nurses, GPs, primary care specialists, nurse practitioners, family carers, and visiting occupational therapists. The workshops lasted an average of 70 minutes.

During the workshops, a facilitator (KW) introduced the appreciative inquiry 4D cycle phases, using the findings from the survey and interviews to guide the conversations about workshop participants’ experiences and suggestions for working well together. Participants acted as co‐researchers to further refine and challenge themes identified from the survey and interviews and themes’ ‘underlying ideas’. They assessed the importance and agreement of what was thought to work and articulated actions that they could take and what needed to be in place. All workshops were audio-recorded, transcribed, and supplemented with written notes for analysis to enable the facilitator to stay focused and ensure that all discussions were captured.

### Data analysis

We used Nvivo 14 to manage the data from the survey, interviews and workshops. We used a thematic approach to map and analyse the data.^21^ Survey free text responses were coded by topic and then grouped thematically. These themes informed the interview prompts and subsequent coding. Interview data were coded, using the appreciative inquiry 4D cycle and the proposed themes were reviewed, redrafted, and elaborated. Themes informed workshops as mentioned above, with workshop participants assessing the importance and agreement of what was thought to work and articulated actions that they could take and what needed to be in place. We then used the findings from all three data sources to populate the ToC model by identifying and refining the themes about key elements of a ToC, namely external factors, activities, strategies, outcomes, and impact.

### Patient and public involvement

A patient and public involvement representative (AA) contributed to the interview data analysis and workshop planning. They reviewed and consulted about the workshop design and materials. They also helped facilitate the discussion in one of the workshops.

## Results

We report on qualitative data from 33 survey free text responses, 15 semi-structured interviews, and eight workshops with a total of 16 participants (Online Supplement Table S1). First, we briefly discuss the findings of each data source; for an overview of the themes identified from survey responses and interviews that were presented during the workshops and contributed to the workshop discussions please see Online Supplement Table S2. We then report on the synthesis of the data to build the Theory of Change.

### Online survey of GP practice staff

The survey was completed by 67 practice staff^20^ of whom 33 provided free text responses. The topics raised concerned: staffing issues, training, digital records and systems, visit preparation and observations, visit necessity, care home variability, communication, and specific GP support. Key themes related to dedicated or available resources for care homes, characteristics of the care home, and communication within the primary care team and with care home staff.

### Interviews with care home and primary care staff

Interview participants discussed social and operational factors that they felt contributed to positive working relationships between care homes and primary care. Examples included two-way communication, psychological safety, or the perception that the benefits of speaking up outweigh the costs to the speaker, and regular meetings. Participants drew on their experiences of the COVID-19 pandemic and the pressures it had caused to describe how mutual trust and working as a team had built a sense of collective endeavour.

*[T]here was a bit of a camaraderie, and a bit of we're all in this together sort of thing. And I think that's why they really appreciated the service what have you, because we were there when they needed us. *(Pharmacist)

Being able to build this trust was characterised as recognising the power dynamics of professionals working with care staff, maintaining two-way communication, providing support, and valuing each other’s work. Teamwork was seen to be only possible when staff were available, contributed their respective expertise, and were well-organised.

Participants discussed the actions and resources that they believed were needed for service improvement and to achieve sustainable relationships. They suggested that the availability of funding and for staff to have time for reviews, training and guidance about deterioration was needed to ensure that care home staff and residents could influence and access primary care support. The discussion reflected policy changes and drew on what had worked well during the pandemic, such as the upskilling of care home staff and primary care staff reassuring and supporting care homes.

*They [care home staff] were woefully underprepared, as were most people, but didn't really have the support around them to make up for that, and we worked very hard with them, to be honest, to try and help them through it…We gave them PPE [personal protective equipment] when they couldn't get hold of it themselves. We went in, and we did training with them on how to don and doff their PPE…Yeah, so we did a lot of work with them on trying to get them through it. *(Paramedic practitioner)

### Workshops with stakeholders

Most workshop participants supported the statements and experiences discussed in the interviews, which were presented in the form of powerpoint slides. Participants agreed that what was reported in the interviews and surveys contributed to positive working relationships between care homes and primary care.

Participants felt that the discussions validated diverse experiences and acknowledged a shared understanding of what constitutes good working relationships and practices.

*[I]t was actually nice to see your slides and other people are thinking the same thing... I think there's the big body of people wanting to do the same things is just how we get sort of put that into action and move things forward. So it was really reassuring to see the things that are on your slides, it was good.* (Nurse working at a large care home)

There was agreement about the need for investment of resources for care homes and primary care to ensure improvement and participants’ suggestions about better working between care homes and primary care. This highlighted a shared recognition of what needs to be in place for success.

The survey and interview findings did not include the family’s involvement in the care of residents. Family carers’ role was stressed by workshop participants as important and reflected family carer participants in the workshops. A participant discussed how family carers have important information regarding the resident’s needs, care and preferences, but their voice is often missing, which can have consequences.

*Where I am, we have a very large, diverse population with different cultural needs, different language needs, mental health needs, and that is not a criticism to anyone… The constant feedback that I'm receiving is they can't speak to the clinicians either in the care homes or GP looking after them in a meaningful way. The patients are therefore getting neglected and they ending up in the hospital sometimes because their conditions have deteriorated quite substantially. *(Family carer)

Family carers argued that family involvement would lead to shared decision-making with care planning and fewer concerns about the residents’ quality of care. Communication with the families was thus seen as critical to ensuring resident preferences were known and factored into decisions.

Table 1 summarises workshop participants’ recommendations for resources and improving working relationships. It was further noted that celebrating and acknowledging good practice should be encouraged. Examples included sharing stories of excellent care or timely referrals with colleagues and residents’ families; recognition from commissioners of good practice; and informally complimenting someone’s good work.

*[W]e could celebrate the things that we're doing that we are communicating well, that and the things that we are doing well. And and…that would like our success even bigger. *(Registered care home manager)

**Table 1.** Recommendations for resources and how to facilitate positive working relationships.

Care homes specific	Protected funds or payment for care home staff undertaking training (e.g. in management of dementia, signs of deterioration, or catheters)
	Investment in technology for the care homes that can share information or be accessible by primary care
	Guidance about when a GP visit is necessary and preparation needed
Primary care specific	Funds to employ GPs and additional primary care staff with appropriate training and skills
	Funding to support regular care home visits by primary care staff
Care home and primary care	Time to set up information-sharing systems and additional technical support to maintain these systems
	Communicate about respective capacity such as the best times to call or visit (or not) and set clear and agreed expectations for everyone’s role
	Opportunities to provide feedback to improve services, review how they work together, share good practice, and celebrate successes

Participants highlighted that too often the focus was on problems and poor care, which was seen to reinforce defensive practices and lack of trust.

### Theory of change for the relationship between care homes and primary care

**Figure 1.**
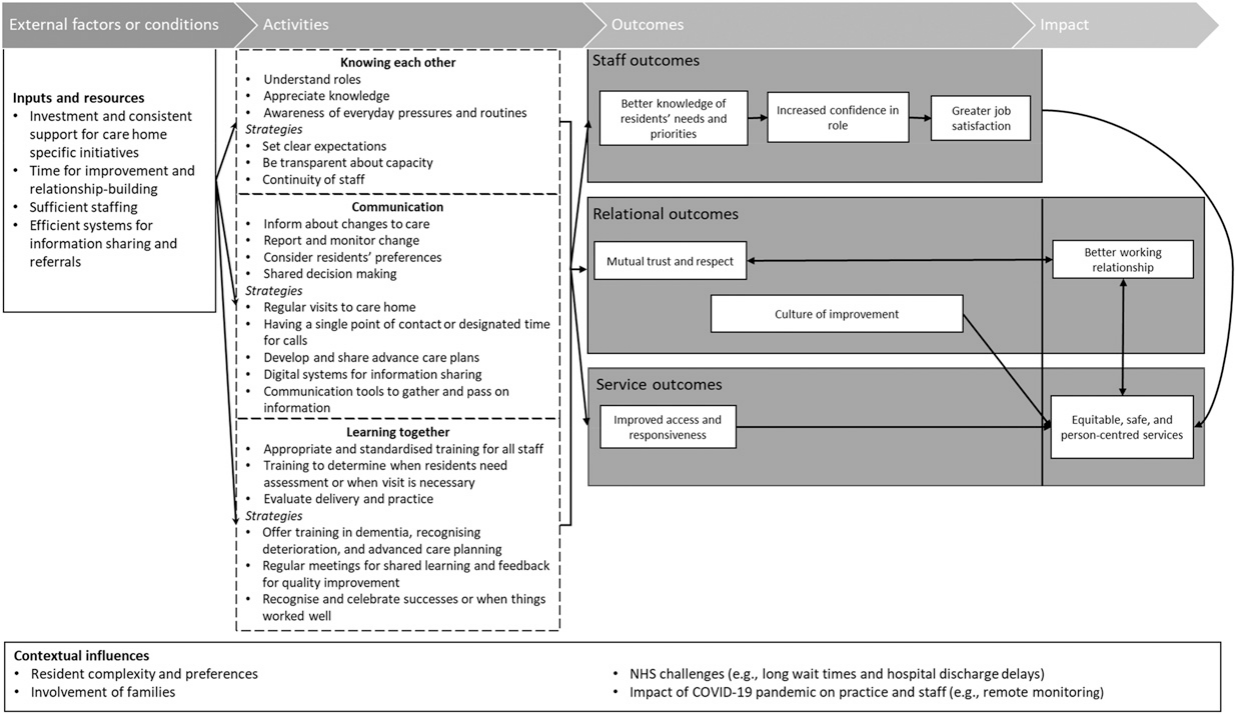
Proposed Theory of Change for the relationship between care homes and primary care.

Figure 1 illustrates the proposed theory of change that we developed for care home and primary care working relationships. We describe the key elements: impact and outcomes, activities, external factors and contextual influences.

The desired impact was working relationships between care homes and primary care that were effective in supporting a person-centred service, recognising that these are linked.

*Just giving something to the residents, and making their lives happy, making sure their health is good. So that's why you need a good rapport with GPs and nurses, and everyone really who deals with their care…The residents, at the end of the day, they need to be prioritised.* (Care home manager)

Outcomes were identified to fall into three categories: staff outcomes, relational outcomes and service outcomes. It was recognised that care home and primary care staff would benefit by having greater job satisfaction and confidence in their roles and an appreciation of each other’s roles in supporting older people.

*I've achieved something, and I've looked after them in their best interests and trying to do my best for them, so it makes you feel good. *(Care home deputy manager)

Having more detailed and regular contact was thought to ultimately lead to residents’ needs and priorities being known and anticipated. Relational outcomes concerned the mutual trust and respect built and a culture that encouraged and supported the review and improvement of services. Planned discussions and opportunities for constructive feedback between care homes and primary care staff offered valuable insights, assessed what was working well, and highlighted best practices. Service-related outcomes were primarily about access and responsiveness to the needs of residents.

Activities or strategies suggested to make outcomes happen were identified as knowing each other, communication, and learning together. The ‘knowing each other’ theme related to actively working to understand and appreciate everyone’s role, especially care home staff’s knowledge of residents and understanding how care homes operate (*“Key is understanding each other’s roles and how we work*.*”* (GP)). Examples include organising GP or other specialist visits to coincide with quieter times in the care home when staff could be available for discussion and review. Also, having clear expectations about people’s respective capacity or what was achievable within existing resources. The need for pragmatism was a recurring point made, especially by primary care staff for whom care home residents were a small part of a pressured workload. Continuity of staff involvement enabled this relationship to build and trust to be developed. However, staff turnover was seen as an ongoing challenge in both health and social care.

The ‘communication’ theme related to how care homes and primary care shared information about residents. This included changes to care, observations of deterioration, monitoring, advanced care planning, and residents’ preferences. Participants discussed how various technologies (email, telephone calls, videoconferencing, and shared record systems) could improve access and responsiveness; this would however require a robust infrastructure. Participants believed that visiting the care home in person was still necessary, especially for GPs, and that it was important to not only assess the resident but to develop positive relationships with the care home staff and residents. The consequences of lack of in-person attendance were often discussed when reflecting on the COVID-19 pandemic.

*It was a struggle, obviously, because they weren't, or couldn't come to the home and visit, so that was a bit hard. But they're very quick to respond by email and telephone calls, really, and … any advice you need, they're there and, yeah, they help me out a lot, actually, the surgery and they're very good. *(Care home deputy manager)

Secondary to this was having a member of care home staff as the single point of contact to strengthen communication, to reduce the risk of information being lost, or not being passed on.

The ‘learning together’ theme related to how activities supporting training or shared learning occurred. Participants focused on those topics that they felt were most likely to be mutually beneficial and enable better care delivery. Suggested topics included dementia, advanced care planning, and end-of-life care. A particular challenge identified by practitioners was the need for standardisation of skills and knowledge to build trust and a shared narrative of what matters. Other areas that were considered to contribute to better outcomes for residents were very specific, such as the ability to determine when a visit was necessary or shared training to support the identification of residents needing assessment.

*In the home with a clear policy in place for contacting the surgeries and being able to provide examination findings for observations such as oxygen saturations, pulse, respiratory, blood pressure, helps the clinicians on the site to make their decisions on the urgency of situations. And also appropriately contacting either the GP via reception and be sent … either paramedics and the rest of the teams, or their GP or 111 when necessary, or 999 when necessary. And also contacting the district nursing, and …so the other community teams, so that they can contact directly.* (GP)

Participants further discussed that they valued using times of review and feedback to learn from shared successes and challenges in working together. Crucially, celebrating when they had worked well or provided exceptional care was seen as strengthening trust and building shared priorities and values.

*[A]ll the feedback can be brought in, and they can improve it on a regular basis. So there can be some encouragement along that side for them, to become more like a learning organisation where they can improve.* (GP)

In an environment where reasons to work together centre around individual residents or patients, this activity was seen as fostering a culture of improvement that went beyond single clinical encounters.

External factors and contextual influences were described by participants to be often outside their control (see also Table 1). Participants identified the need for investment to release staff to deliver care home-specific policies or services (e.g., EHCH framework), along with adequate staffing, including additional roles in primary care to support GPs, with access to appropriate training and information technology infrastructure. The heterogeneity of residents’ needs and their complexity, such as nearing the end of life, dementia, frailty, and polypharmacy, and the crucial need to involve families were additional to the well-documented challenges of high patient demand in primary care.

For some, the lasting impact of the COVID-19 pandemic was that it created a sense of ‘we’re in this together’. Care home staff were recognised by primary care and other visiting professionals for their knowledge and commitment to residents’ care as they acquired new skills and responsibilities. A perceived post-pandemic increase in the use of online communication and information technology by care homes had, for some, led to regular calls or virtual ward rounds. This was seen as a positive experience although loss of staff and ongoing recruitment challenges for the sector mitigated some of the advantages arising from greater use of digital technology in many care homes.

## Discussion

This paper offers a granular account of what could improve and sustain care homes and primary care working relationships for the benefit of residents in one region in England. We combined different sets of data to develop a Theory of Change, which offers a resource to review current practice and how the organisation and delivery of services are more (or less) likely to enable positive care home and primary care working relationships.

Our findings support previous evidence on the need for trust, information sharing, continuity of staff, recognition and understanding of each other’s role, and communication to improve cross-sector working.^5,19,23^ How primary care works with long-term care resonates with the theories of the complexities and challenges of inter-organisational collaboration, developed from various countries, and the need for significant time and resources to address these challenges, such as staff differing backgrounds, power dynamics, poor communication, and complex and contradictory regulatory environments.^23^

In this study, we developed a Theory of Change using evidence from accounts of successes and improvement rather than the well-documented problems and challenges. Using an appreciative inquiry approach, our findings reflect experiences of what is seen to work well to identify key principles of working together and clarify what strategies may be achievable. Workshop participants found the focus on what works helpful in structuring their reflection and reframing ongoing challenges. An important finding was that workshop participants valued the (rare) experience of care home and primary care staff comparing their practice and its achievements with those of colleagues regionally and in other parts of the country. Appreciative inquiry has been successfully used in quality improvement collaboratives between care homes and NHS services.^24^ In our study, it facilitated staff working toward a shared goal of caring for residents from different organisations or sectors.^19^

The study took place at the beginning of the rollout of the Enhanced Health Care in Care Homes (EHCH) framework in England.^3^ This framework assumes that partnership and aligned working between care homes and primary care is both important and possible. Early evidence suggests that a shift towards GP practices working with fewer care homes may lead to improved relationships. However, it is unclear what was driving these changes and the role of the COVID-19 pandemic as a catalyst for change.^20^ The ToC suggests key drivers such as greater resources to create time to work together and additional staff^9^; systems for regularly sharing and discussing information, for example, virtual ward rounds; continuity from a clinical lead (as stipulated in the EHCH framework); and ongoing workforce development. The ToC could be used to structure future evaluations of the EHCH framework, by assessing how it enables the inputs and resources for positive care home and primary care working relationships.

The work presented here also offered an opportunity to reflect on COVID-19 pandemic experiences and learning. During this exceptional time, the collaboration between health and social care was tested.^6^ Creative or new ways of working were enacted, yet the sustainability of these new ways of working is yet to be determined.^25^ The pandemic has further highlighted the need for greater health and social care integration. In England, the creation of Integrated Care Systems in 2022^26^ and the 2023 primary care access plan^27^ aim to enable better cross-sector working and information sharing.

Participants in this study were various practitioners and representatives of service users, and their discussions often focused on the individual resident or care home encounters. Overall, there was very little acknowledgement of how these system-wide organisational changes affected their practice, besides the changes due to the pandemic. For example, the role of primary care in care homes is changing, with a wider range of practitioners likely to be involved.^9^ Unlike earlier studies, we included these practitioners to better understand their role and contribution to the working relationship between care homes and primary care. Future research should consider their part and impact on how care is delivered. Similarly, the rapid digitalisation of social care (e.g., digital social care records) and the assumption that data will be key in facilitating decision-making and service development were raised, but not developed as key resources or tools for partnership working.^28^ This study, undertaken in a time of ongoing organisational turnover and rapid change, highlights the value of retaining and supporting what is already working well and, preserving organisational memory, to foster cross-sector working. Lessons learned and recommendations are likely to be transferable to other settings such as proactive management of multiple long-term conditions and the ageing population; both take on greater policy prominence in the UK and internationally as the current evidence is lacking.^29^

### Strengths and limitations

A principal strength of this study was its multi-method approach. By using data from different sources and methods, the findings could be combined to provide a better understanding and inform the ToC. Another strength was the sampling strategy to represent different care models, informed by our survey results.^20^ By interviewing both care home and primary care staff, the interview topics could be explored from different perspectives, increasing the trustworthiness of the study. The use of workshops to identify what practitioners valued and shared outcomes of interest tested the relevance and transferability of the findings.

The ToC proposed in this study is not comprehensive and its development was constrained by the low response rate and small sample. Our study did not distinguish between different types of care homes, for example, those with or without nursing onsite or with a dementia speciality, and their differing needs. Other work has suggested that care homes without onsite services have different ways of using NHS services.^30^ This study was also limited as it relied on mostly staff participant responses and the residents’ voice is missing. We did invite residents to take part but none did. Additional work could refine and develop the ToC to inform how to support primary and long-term care partnerships.

## Conclusions

This multi-method study presents the development of a ToC of care home and primary care working that offers several dimensions of good practice and provides insight into areas for action. It provides evidence of how learning from experiences during the COVID-19 pandemic has been incorporated into routine primary care practice, ways of working with care homes and the importance of retaining these benefits.

## Supplemental Material

Supplemental Material - Care homes and primary care in England working together: A multi-method qualitative study Supplemental Material for Care homes and primary care in England working together: A multi-method qualitative study by Krystal Warmoth, Alex Aylward and Claire Goodman in Journal of Health Services Research & Policy
