# Supplementary material for: Care homes and primary care in England working together: A multi-method qualitative study
Source: J Health Serv Res Policy. 2024 Dec 13;30(2):127–35. doi: 10.1177/13558196241306607 (PMC11877972; doi:10.1177/13558196241306607)
Supplement: Supplemental Material - Care homes and primary care in England working together: A multi-method qualitative study [file sj-pdf-1-hsr-10.1177_13558196241306607.pdf]

Online Supplement

Contents

Interview Topic Guide 2

Table S1. Overview of study participants 5

Table S2. Themes identified from interviews and survey responses and presented during coproduction workshops 6

Interview Topic Guide

TOPIC GUIDE – STAFF

Introduction

Good morning/afternoon. My name is XXXXX. I am going to ask you some questions about the relationship between care homes and primary care.

Before we start, let me explain a bit about what we are going to do, because it may be a little different from what you have done in the past. I will be asking you questions about when you have experienced things working at their best and what made it happen that way because the more, we know what makes things work well, the more we can improve our performance in the future.

So, I won't be asking about problems or searching for ways to fix them. Instead, I need to hear about what is working well, and the factors that make things work well.

Again, I want to remind you that your responses will be kept confidential and you do not have to answer any question if you do not wish to. You can also stop the interview at any time.

Before we start, I want to check that you are happy to be interviewed and for the interview to be recorded.

Interview

**AI phase**	**Question**	**Probes**
Discovery	To start, please tell me about your role in the relationship between care homes and primary care (GP).	• What do you value most about this work?<br>• What are you most proud of?<br>• What strengths do you bring to this role?

	Can you tell me about a successful experience, or when you have experienced things working at their best, relating to the relationship between care homes and primary care (GP) during the pandemic?	• What made it possible?<br>• What was your contribution?<br>• How did you feel?<br>• What exact strengths were you and your colleagues showing?<br>• What made it so exceptional?<br>• What was the impact?<br>• What lessons can we take going forward in future collaborations?<br>• How can this experience help achieve similar successes moving forward?<br>• What strengths from this experience could you develop further for continued positive outcomes?
Dream	Imagine your ideal future for this relationship. What does it look like?	• What images come to mind?<br>• What three wishes do you have for changing this relationship?<br>• What do your teams look like?<br>• How do your communications flow?<br>• How are you interacting with one another and with residents and their family carers?<br>• What do our meetings look like?
Design/Destiny	Picturing this ideal future for the relationship. What actions need to be taken to make this happen?	• What resources need to be in place?<br>• Who needs to be involved?<br>• What would be the biggest achievements?<br>• What should be prioritised?<br>• What do you need to sustain the development?

Closure

Is there any other information regarding your opinions or experiences that you think would be useful for me to know?

Thank you very much for meeting with me. Your time is very much appreciated and your comments have been very helpful.

Table S1. Overview of study participants

Job/role	Number of participants		
	Survey	Interview	Workshops
GP	24	6	1
Nurse practitioner or registered nurse	4	1	3
Pharmacist	1	1	
Paramedic practitioner	1	1	
Primary care specialist			1
Other primary care staff	2	1	
Care home manager or director		2	6
Care home deputy manager		3	1
Care home clinical lead			1
Senior carer			1
Family carer			1
Other community specialist			1
Total	33	15	16

Table S2. Themes identified from interviews and survey responses and presented during coproduction workshops

Appreciate Inquiry phase	Themes	Supporting quote
Discover	Two-way communication	And that is sort of necessary, isn't it? But whoever it is, they need to have the skills and the confidence themselves, the staff at the home, to need to trust that person and there needs to be that two-way communication. (female GP supporting 2 care homes)
	Regular meetings or calls	A regular ward round that we have done for those two homes, with a nominated GP for each, and that has always helped. Both - it helps the staff and the patients at those homes, but it also helps us a little bit in managing the work that comes out of the home. (female GP supporting 2 care homes)
	Well-organised teams	It worked very well with one of the care homes, and the other care home actually was far better managed and they had less vulnerable patients, so less demand, and slightly more capable seniors on-site who could make, in my view, more better decisions around the care of the patients that didn't need us, our input all the time. But the other care home has got more vulnerable patients, including some on one-on-one care, and I still do a ward round there once a week. (female care home manager)
	Feeling part of the team or valued	actually, we all - there was a bit of a camaraderie, and a bit of we're all in this together sort of thing. And I think that's why they really appreciated the service what have you, because we were there when they needed us (male pharmacist supporting 5 care homes)
	Understanding roles and expectations	I think it is, just like I said at the start, having that boundary and knowing where your practice lies, and not trying to be dictatorial to them, or more supreme because I'm a nurse, and I've got all this information and training, and you're just a carer. (female nurse practitioner supporting 2 care homes)
	'Knowing the residents'	I think it means that the patients that we look after there, we just have perhaps that additional level of knowledge of them, of their needs. (female GP supporting 2 care homes)
	Trusted point of contact	But certainly having the paramedic there for us, is… Or a nurse practitioner, for that matter. And having their person - having their own continuity of care, I think is the key thing. (male GP supporting 3 care homes)
Dream	Visiting still happens	It would be them coming in, literally into the home, either weekly or fortnightly action to see the residents and sort of like… You can - we're quite a small home, so they can walk around and just pass their eye over. So, yeah, definitely more coming into the home would be good. (female deputy manager of a care home)
	Time to plan and work together	I think it's just being allocated the time, is the most important thing for all of us. Having a set, appropriate time that's good for the care home and good for us, I think that's the major thing, and to keep it in this ideal future would be to have a good amount of time. Ideally, a whole morning would be better, but two hours used to be sufficient. And I was quite lucky, I think some care homes don't - the clinician doesn't get that long. (female nurse practitioner supporting 2 care homes)
	Good information collection and sharing	everything has got a paper trail, so everything you do, every GP visit, every GP phone call is all documented. So when you think - go back to think, oh, when did so-and-so have that UTI, you've got it documented, so everything has to be communicated and, like I say, a paper trail of everything (female care home manager)
	Prompt responses	If I was having issues with one of the residents, I'd email them and they

Appreciate Inquiry phase	Themes	Supporting quote
	to queries and calls	respond very, very quickly and very promptly to emails that we send. It was a struggle, obviously, because they weren't, or couldn't come to the home and visit, so that was a bit hard. But they're very quick to respond by email and telephone calls, really, and … any advice you need, they're there and, yeah, they help me out a lot, actually, the surgery and they're very good. (female care home deputy manager)
	Holistic and compassionate resident-centred care	Just giving something to the residents, and making their lives happy, making sure their health is good. So that's why you need a good rapport with GPs and nurses, and everyone really who deals with their care…The residents, at the end of the day, they need to be prioritised. (female care home manager)
	Satisfied and proud of role	It's like I've achieved something, and I've looked after them in their best interests and trying to do my best for them, so it makes you feel good. (female care home deputy manager)
Design/Destiny	Investment (funds and time)	More time and access for clinicians to have allocated time for the additional reviews, or the third-party care teams to have the ability to provide that service…Time. Money. (Reception manager and data administrator for practice supporting 1 care home)
	Training	They could be educated on picking up early changes in patients, when the vitals change or when there's a weight loss, which they do. They do [deterioration] scores on these patients on a weekly basis, and they report to us if there is a change. So more kind of proactive work once a week, we've been doing. (male GP supporting 3 care homes)
	Additional supportive roles	But certainly having the paramedic there for us… Or a nurse practitioner, for that matter. And having their person - having their own continuity of care, I think is the key thing. And then having access to other resources, and social services, and that sort of thing. (female GP supporting 4 care homes)
	Mutual respect	I've been here for two years and, yeah, just building those working relationships, just this comes down to that respect thing, doesn't it?... I think it's just being friendly and making them feel welcomed when they come here. They're a part of our home, just as much as we are and we rely on them, so just respect. (female care home deputy manager)
	Guidance when visits and referrals are needed	In the home with a clear policy in place for contacting the surgeries, and being able to provide examination findings for observations such as oxygen saturations, pulse, respiratory, blood pressure, helps the clinicians on the site to make their decisions on the urgency of situations. And also appropriately contacting either the GP via reception, and be sent … either paramedics and the rest of the teams, or their GP or 111 when necessary, or 999 when necessary. And also contacting the district nursing, and …so the other community teams, so that they can contact directly. (male GP supporting 3 care homes)
	Care planning together (incl. end of life discussions)	I've asked our care homes, once a resident has settled in, after about six weeks, they're buddied, so whoever the member of staff is that's more affiliated, to get to know that resident and their families, they're the best place people to start those discussions about end-of-life planning, and have you considered? And it's a good opportunity to say that now this next journey in your life, you're a resident here, this is now your home, let's make sure that we honour all of your wishes and make sure they know what those choices are. So our care homes are very good at doing that now, and they'll come to me and say, we've completed an advanced care plan with this patient. (female GP supporting 3 care homes)
